# Evaluation of lung epithelial lining fluid concentrations of lascufloxacin against *Streptococcus pneumoniae* in a hollow-fiber infection model

**DOI:** 10.1038/s41429-025-00826-7

**Published:** 2025-05-07

**Authors:** Haruka Nakagawa Kamura, Tetsuo Yamaguchi, Toshihiro Kasama, Yukitaka Hayashi, Masakaze Hamada, Kazuaki Matsumoto, Ryo Miyake, Yoshikazu Ishii

**Affiliations:** 1 Microbial Genomics and Ecology, Center for the Planetary Health and Innovation Science (PHIS), The IDEC Institute, Hiroshima University, Hiroshima, Japan; 2 Department of Microbiology and Infectious Diseases, Toho University School of Medicine, Tokyo, Japan; 3 Department of Bioengineering, Graduate School of Engineering, The University of Tokyo, Tokyo, Japan; 4 Division of Pharmacodynamics, Faculty of Pharmacy, Keio University, Tokyo, Japan

**Keywords:** Antibiotics, Phenotypic screening

## Abstract

Lascufloxacin (LSFX) achieves concentrations in epithelial lining fluid (ELF) that are more than 15 times higher than those in the bloodstream, making it a promising candidate for respiratory and otorhinolaryngological infections. These concentrations were replicated using the Hollow-Fiber Infection Model and demonstrated bactericidal efficacy of LSFX against levofloxacin-sensitive and -resistant *Streptococcus pneumoniae*. The study confirms that LSFX’s elevated concentration in ELF plays a significant role in its bactericidal activity.

Lascufloxacin (LSFX) is a novel quinolone antibiotic characterized by its concentration in lung epithelial lining fluid (ELF), which is 15.0–22.4 times higher than in blood and 18.5–56.4 times higher in alveolar macrophages [1, 2]. These properties make LSFX a potential therapeutic option for treating respiratory and otorhinolaryngological infections.

The hollow-fiber infection model (HFIM) is an advanced in vitro system used to evaluate the efficacy of antimicrobial treatments against infections under controlled conditions. The model uses hollow-fiber modules that simulate human infection sites by replicating antimicrobial exposure and pathogen growth at concentrations close to physiological conditions [3], thus offering multiple applications in antimicrobial drug assessment and breakpoint determinations [3, 4].

In this study, to evaluate the efficacy of antimicrobials against *Streptococcus pneumoniae* using the HFIM, a medium that promotes *S. pneumoniae* growth and a venting device to eliminate air bubbles in the hollow-fiber module were developed. The efficacy of LSFX was assessed by simulating LSFX human plasma and ELF concentrations with both levofloxacin (LVFX)-sensitive *S. pneumoniae* TUM23169 (MIC 1 mg l^−1^) and LVFX-resistant *S. pneumoniae* TUM23133 (MIC > 4 mg l^−1^), isolated from respiratory specimens [5, 6]. These strains were stored in LB medium with 15% glycerol at −80 °C. Cation-adjusted Mueller-Hinton broth (CAMHB), Mueller-Hinton agar (MHA), and defibrinated sheep blood were obtained from Becton, Dickinson and Company (Franklin Lakes, NJ) and Nippon Bio-Supp.Center (Tokyo, Japan), respectively. LSFX was provided by KYORIN Pharmaceutical Co., Ltd. (Tokyo, Japan).

The microbroth dilution and agar dilution methods for *S. pneumoniae* followed the Clinical and Laboratory Standards Institute document [7], using cation-adjusted Mueller-Hinton broth with 5% sterile lysed horse blood (LHB-CAMHB) and Mueller-Hinton agar with 5% sterile defibrinated sheep blood (SB-MHA). However, when 5% LHB-CAMHB was used in the HFIM, testing had to be discontinued due to biofouling of the hollow-fiber membrane by the blood components. Consequently, a blood substitute without protein components, Chemically Defined Medium (CDM)-HD (FiberCell Systems Inc., MD), was added to reach a 10% concentration. As a result, 5% LHB-CAMHB demonstrated a growth rate of *S. pneumoniae* similar to that of 10% CDM-CAMHB when incubated under identical shaking culture conditions, being 10-fold (10^9^ CFU ml^−1^) or more than the unsupplemented CAMHB (approximately 10^8^ CFU ml l^−1^) after 8 h. When the unsupplemented CAMHB was used, the bacteriolysis phenomenon was observed from after around 6 h in a shaking culture (data not shown).

The MIC values for LSFX against TUM23169 and TUM23133 were 0.03 mg l^−1^ and 0.25 mg l^−1^, respectively. using either 10% CDM-CAMHB or 5% LHB-CAMHB. Additionally, the MIC values for LSFX against TUM23169 and TUM23133 determined by the agar dilution method were 0.06 mg l^−1^ and 0.25 mg l^−1^, respectively. The frequency of mutant strain emergence against LSFX was investigated using test strains at 10^9^ CFU ml^−1^, and no mutant strains were identified, confirming that they were not high-frequency mutant-emerging strains. LSFX quantification was performed according to the method previously reported by Furuie et al. [8]. The HFIM was conducted as described in prior studies [9].

We commissioned the fabrication of a venting system from a machined aluminum block, as shown in Fig. 1 (Niigata Co., Ltd, Kanagawa, Japan). Since aluminum is prone to corrosion from the medium components, the block was anodized via a wet process after cutting (Sankyo Alumite Co., Ltd, Tokyo, Japan). Without the venting device, the two *S. pneumoniae* strains used as test strains in this study caused the hollow-fiber module to fill with air bubbles, but no bubbles were observed when the venting device shown in Fig. 1 was installed. The HFIM constructed for this study operated continuously for seven days, and no air bubbles were observed in the hollow-fiber module.

**Fig. 1:**
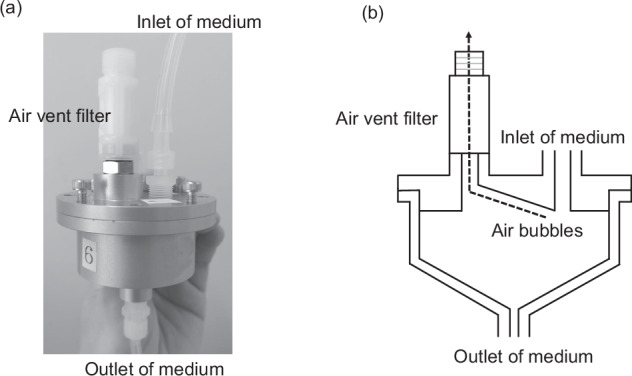
Air Vent System Created by Anodized Aluminum Processing Following Aluminum Machining. This air vent system, produced through anodizing following aluminum machining, consists of a main body and a lid, as shown in the external photograph, and is assembled by fastening these two parts together with screws (**a**). Designed to be autoclavable, the system includes a vent filter and is equipped with a silicone gasket between parts to prevent medium leakage. The internal structure is shown in (**b**). Medium discharged from the hollow fiber module enters from the upper inlet, and any air bubbles in the medium move along an incline within the air vent device, where they are expelled through an air vent filter (pore size 0.2 mm). If any bubbles remain in this inclined section, they are removed by an additional incline created up to the medium outlet. The medium, now free of bubbles, returns to the extracapillary space of the hollow fiber module through the lower outlet

While detailed data are not shown, a chemostat model was employed to simulate human plasma concentrations and evaluate the effects of LSFX on *S. pneumoniae* strains TUM23169 and TUM23133. The chemostat model analysis demonstrated that the bacterial counts of TUM23169 declined below the detection limit within 6 h, whereas TUM23133 remained detectable even after 24 h. Where the pharmacokinetics of LSFX within ELF were reproduced, bactericidal effects were observed for both strains, with Δlog CFU_0–24_ < −3.74 against TUM23169 and Δlog CFU_0–24_ < −4.48 against TUM23133. Under blood pharmacokinetic conditions, a bactericidal effect (Δlog CFU_0–24_ < −4.31) was noted for TUM23169, whereas a bacteriostatic effect (Δlog CFU_0–24_ = −1.31) was observed for TUM23133.

To further investigate, we employed the HFIM to simulate LSFX concentrations in ELF and evaluate its impact on TUM23169 and TUM23133. The bacterial count of TUM23169 decreased from an initial 6.01 log_10_ CFU ml^−1^ to below the detection limit (100 CFU ml^−1^) within 4 h of LSFX administration, and no re-growth occurred through 168 h (Fig. 2a). For TUM23133, the initial bacterial count of 6.22 log_10_ CFU ml^−1^ similarly decreased to the detection limit within four hours, with no re-growth observed over 168 h.

**Fig. 2:**
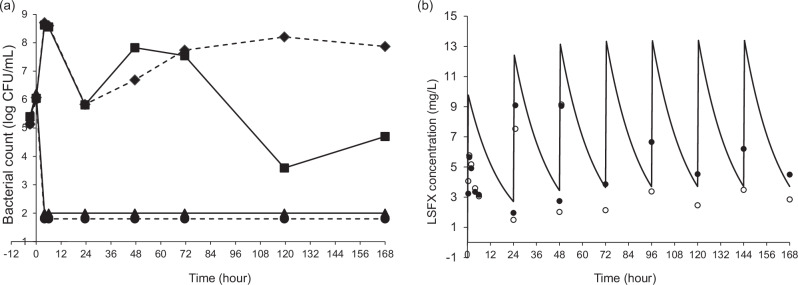
Simulated concentration of lascufloxacin (LSFX) in human epithelial lining fluid (ELF) over seven days and its effect on bacterial counts of levofloxacin-susceptible and -resistant *Streptococcus pneumoniae*: bacterial count (**a**) and measured LSFX concentrations (**b**). The solid and dashed lines in (**a**) represent the trends in the bacterial counts of levofloxacin-resistant (TUM23133) and -sensitive (TUM23169) *S. pneumoniae*, respectively. The squares and diamonds correspond to the groups without LSFX addition, while the triangles and circles indicate the trends in bacterial counts simulated under the ELF concentrations of LSFX. The detection limit for all measurements is 100 CFU ml^−1^. In (**b**), measured LSFX concentrations for both *S. pneumoniae* TUM23169 (open circles) and *S. pneumoniae* TUM23133 (closed circles) remained below theoretical values across all sampling points

The dynamics of LSFX concentrations in ELF were simulated by the HFIM for seven days (Fig. 2b), to model the potential use of LSFX for treating respiratory infections caused by *S. pneumoniae*, including LSFX-reduced susceptible strains. Moving forward, these data demonstrate the importance of incorporating PK/PD analysis data based on pharmacokinetic parameters obtained from infected tissues when establishing breakpoints.

In this study, a novel culture medium was proposed for use with *S. pneumoniae* as a test strain, a target not previously addressed by the HFIM, yielding promising results. This suggests that the number of strains suitable for HFIM could be expanded by modifying the culture medium. In today’s climate, where there is increasing pressure to limit animal experimentation, expanding the range of bacterial species that can be tested using the HFIM could contribute to reducing the need for animal studies.

During HFIM operation, air bubble-induced fouling in the hollow-fiber module can occur, depending on the species tested and the combination of the species and antimicrobial agent [10], which can force experiments to be interrupted. In HFIM experiments, we observed the occurrence of air bubbles within the hollow fiber cartridge, which depended on the bacterial species and the combinations of bacteria and antimicrobial agents. The *S. pneumoniae* strain used in this study is characterized by autolysis during division and growth, which may contribute to the increased likelihood of air bubble formation. Accumulation of air bubbles in the ECS reduces the volume of the medium, potentially affecting drug concentrations and bacterial growth. Furthermore, as the medium volume decreases, the ECS can eventually become entirely devoid of medium, rendering the continuation of the experiment impossible. To prevent this issue, we attached a three-way stopcock to the internal circulation port of the ECS and removed the bubbles with a syringe. However, this procedure frequently led to contamination with unwanted microorganisms.

The limitations of this study are as follows. First, the measured antibiotic concentrations were lower than the theoretical values, suggesting that the in vivo ELF concentrations [2] were not reached. This discrepancy may be attributed to the possibility that LSFX was absorbed by the hollow-fiber membrane or to factors related to the components used in the system [11]. To address this issue, future improvements to the system components, including the hollow fiber membrane, are necessary. Second, only one strain each of levofloxacin-sensitive and -resistant *S. pneumoniae* was tested. Future studies should incorporate a broader range of bacterial species and strains to further assess LSFX concentrations in ELF. Third, the low initial inoculum size may have affected bactericidal efficacy and the development of resistance. We utilized a spectrophotometer to measure turbidity and estimate the approximate bacterial count. However, adjusting the bacterial count using this method proved challenging for *S. pneumoniae*. Consequently, the measured bacterial count in this study was lower than anticipated. Therefore, standardization of the initial inoculum size in chemostat models or HFIM studies is essential.

In conclusion, the development of a novel medium suitable for culturing *S. pneumoniae* and a bubble removal device has made it possible to evaluate antibiotics against *S. pneumoniae* using HFIM, and this system can mimic the pharmacokinetics of antibiotics in tissues, which is also useful for setting clinical breakpoints. Furthermore, LSFX showed strong bactericidal activity against *S. pneumoniae* at the concentrations observed in ELF. HFIM can be used to set clinical breakpoints by considering the pharmacokinetic properties of antibacterial agents that accumulate in specific sites, such as LSFX.
