# Correction: Evaluation of lung epithelial lining fluid concentrations of lascufloxacin against *Streptococcus pneumoniae* in a hollow-fiber infection model

**DOI:** 10.1038/s41429-025-00837-4

**Published:** 2025-06-16

**Authors:** Haruka Nakagawa Kamura, Tetsuo Yamaguchi, Toshihiro Kasama, Yukitaka Hayashi, Masakaze Hamada, Kazuaki Matsumoto, Ryo Miyake, Yoshikazu Ishii

**Affiliations:** 1 Microbial Genomics and Ecology, Center for the Planetary Health and Innovation Science (PHIS), The IDEC Institute, Hiroshima University, Hiroshima, Japan; 2 Department of Microbiology and Infectious Diseases, Toho University School of Medicine, Tokyo, Japan; 3 Department of Bioengineering, Graduate School of Engineering, The University of Tokyo, Tokyo, Japan; 4 Division of Pharmacodynamics, Faculty of Pharmacy, Keio University, Tokyo, Japan

**Keywords:** Antibiotics, Phenotypic screening

Correction to: *The Journal of Antibiotics*

10.1038/s41429-025-00826-7, published online 07 May 2025

In this article, the Conflict of Interest statement was incorrectly published as “The authors declare no competing interests.” but should have been “This study was funded by KYORIN Pharmaceutical Co., Ltd.”. The original article has been updated.

